# The influence of congenital and developmental cataract surgery on the ocular surface in a six-month follow-up prospective clinical study

**DOI:** 10.1186/s12886-022-02446-3

**Published:** 2022-05-13

**Authors:** Xiaolei Lin, Hongzhe Li, Xiyue Zhou, Xin Liu, Fan Fan, Tianke Yang, Yi Luo

**Affiliations:** 1 Eye Institute and Department of Ophthalmology, Eye and ENT Hospital, Fudan University, Shanghai, China; 2 Shanghai Key Laboratory of Visual Impairment and Restoration, Fudan University, Shanghai, China

**Keywords:** Congenital/developmental cataract surgery, Tear film stability, Meibomian gland function, Meibomian gland morphology, Dry eye

## Abstract

**Background:**

The purpose of this study was to identify changes in tear film function and meibomian gland function in children after congenital/developmental cataract surgery.

**Methods:**

This study enrolled 16 eyes of 16 congenital/developmental cataract patients (mean age: 8.05 ± 1.43 years) who underwent cataract surgery and 16 eyes of 16 normal volunteers (mean age: 8.31 ± 2.18 years). Clinical assessments were conducted preoperatively and at 1 week, 1, 3 and 6 months postoperatively. Symptom questionnaires, non-invasive tear film break-up time, tear meniscus height, corneal fluorescein staining, lid margin abnormality, meibomian gland expressibility, and meibography were assessed.

**Results:**

The ocular symptom score was significantly higher in congenital/developmental cataract patients compared to normal controls during the 5 visits (*P* = 0.009). And the average non-invasive tear film break-up time was significantly lower in congenital/developmental cataract patients compared to normal controls (*P* = 0.017). The first non-invasive tear film break-up time and average non-invasive tear film break-up time were lowest at 1 month postoperatively compared to baseline levels (*P* = 0.008 and *P* = 0.012, respectively). The lid margin score of the upper eyelid was significantly higher in congenital/developmental cataract patients compared to normal controls at 1 week postoperatively (*P* = 0.027). The meibum expressibility score decreased significantly during the 5 visits (*P* = 0.024). No significant difference was observed in meibomian gland tortuosity, meibomian gland width, meibomian gland area and meibomian gland length between the congenital/developmental group and normal controls preoperatively and at 6 months postoperatively (*P* > 0.05).

**Conclusion:**

Tear film stability and meibomian gland function are worsened transiently after congenital/developmental cataract surgery without accompanying meibomian gland morphological changes.

## Background

Congenital/developmental cataract is one of the leading causes of treatable blindness in children. Effective treatment without delay will significantly prevent the occurrence of stimulus deprivation amblyopia and improve the visual quality and life quality of patients. For most congenital/developmental cataract patients, surgery is the only effective treatment that will have an obvious improvement on the visual outcome [1]. Although the techniques of congenital/developmental cataract surgery have evolved and been automated [2], the complications, such as glaucoma and ocular surface damage, remain common, threatening vision quality after surgery.

Dry eye is one of the most common complications after cataract surgery and is associated with several mechanisms causing significant clinical problems, such as corneal melting, severe inflammation or epitheliopathy refractory to medications. During congenital/developmental cataract surgery, some of the corneal nerves are impaired, which decreases corneal sensitivity [3]. It will also affect tear secretion and eyelid blinking, which are triggered by corneal nerve reflexes [4]. Furthermore, surgery-associated inflammation and the use of topical medications after surgery are unavoidable. Moreover, postoperative inflammation in young children tends to be more substantial compared to adults [5]. Therefore, cataract surgery may be a risk factor for tear film dysfunction in congenital/developmental cataract patients.

Meibomian gland dysfunction (MGD) is the leading cause of evaporative dry eye and is developed by obstructed meibomian glands with or without qualitative or quantitative changes of the meibum [6]. Recent studies found that meibomian gland morphological changes, including meibomian gland atrophy and meibomian gland tortuosity, could begin in children without any symptoms [7, 8]. Various ocular and systemic factors have been reported to influence meibomian gland morphology in children, including a higher body mass index (BMI) [7], blepharokeratoconjunctivitis [9], digital device usage [10], and environmental aggravation [8]. However, the effect of congenital/developmental cataract surgery on meibomian gland function has not been investigated.

The purpose of this study is to determine changes in tear film function and meibomian gland function in children after congenital/developmental cataract surgery.

## Materials and methods

### Study population

This prospective study was performed at the Eye and ENT Hospital of Fudan University, Shanghai, China. Written informed consent was obtained from the patients and their guardians before their inclusion in the study. This study was approved by the Investigational Review Board of the Eye and ENT Hospital of Fudan University in Shanghai, China. All the procedures were in compliance with the tenets of the Declaration of Helsinki.

Participants were required to be between 6 and 15 years old. Patients who had a history of ocular trauma or surgery, ocular inflammation, contact lens wearing, or any other disease known to influence the tear film were excluded from the study. The patients with the coexisting ocular disease which may influence the ocular surface were also excluded, such as microcornea, microphthalmos, posterior lenticonus, iris hypoplasia, aniridia, iridocorneal synechiae, and Peters anomaly.

A total of 16 congenital/developmental cataract patients (5 bilateral and 11 unilateral patients; 5 girls and 11 boys; mean age: 8.05 ± 1.43 years; range: 6–12 years) and 16 normal controls (5 girls and 11 boys; mean age: 8.31 ± 2.18 years; range: 6–14 years) were included in the study between July 14, 2020, and January 28, 2021. For the children with bilateral cataract, only the right eyes were included. The normal controls were the fellow eyes of unilateral cataract patients, including 11 unilateral congenital/developmental cataract patients and 5 unilateral traumatic cataract patients.

### Study protocol

All the patients underwent cataract surgery under general anesthesia using the Millennium Microsurgical System (Alcon, Fort Worth, TX, USA) and the 25-gauge microincision vitrectomy system by the same surgeon (Y.L.). The surgical procedures for cataract removal mainly include making a scleral tunnel incision, anterior continuous curvilinear capsulorhexis, and cataract removal by aspiration device. Then a one-piece foldable intraocular lens (IOL) was implanted into the capsular bag primarily. Posterior capsulotomy and anterior vitrectomy were performed for all cataract patients. The scleral tunnel incision was closed with a 10–0 nylon suture. At the end of the surgery, dexamethasone (2.5 mg) was injected subconjunctivally and eye ointment containing tobramycin 0.3% and dexamethasone 0.1% was applied. The operative process was uneventful for all patients. And there were no complications intraoperatively or postoperatively for all the patients, including posterior capsule ruptures, intraocular pressure rises, and severe inflammation.

Postoperatively, prednisolone acetate ophthalmic suspension 1% was administered 4 times daily for 14 days. And cycloplegic eye drops with 0.5% tropicamide plus 0.5% phenylephrine were used 3 times daily for 7 days, followed by 1 time every night before bed for the next 21 days. Topical moxifloxacin 0.5% was applied 4 times daily for 14 days.

A series of tear film and meibomian gland function examinations were conducted preoperatively, at 1 week postoperatively, and at 1, 3 and 6 months postoperatively. Normal controls were also performed 6 months of follow-up for the same examinations.

Clinical measurements were performed in the following order: symptom questionnaires, tear meniscus height (TMH), noninvasive breakup time (NIBUT), corneal fluorescein staining (CFS), lid margin abnormality, meibomian gland expressibility and meibography.

### Subject examination

The 14 dry eye related ocular symptoms were asked of all participants, including ocular fatigue, discharge, foreign body sensation, dryness, uncomfortable sensation, sticky sensation, pain, epiphora, itching, redness, heavy sensation, glare, excessive blinking, and history of chalazion or hordeolum. And the score ranged from 0 to 14.

The results of TMH, NIBUT and meibography were obtained by Keratograph 5 M (K5M; Wetzlar, Germany). The central TMH of the lower eyelid was assessed 5 s after blinking, and the average value of 3 measurements was recorded.

Meibography was also obtained by Keratograph 5 M. Images of both eyelids were captured and used for meiboscore calculation and further meibomian gland morphological analysis. Meiboscores were graded from 0 to 3 for each eyelid using the method described by Arita R [11]. Meibomian gland tortuosity was calculated by the method published previously using VIA 3 software (http://www.robots.ox.ac.uk/∼vgg/software/via/) [12, 13]. The meibomian gland area was the sum area of the central 8 meibomian glands of both eyelids. The meibomian gland length was the minimum external rectangle of the meibomian gland, as has been shown in our previously published paper [13]. Finally, the meibomian gland width was the ratio of the meibomian gland area to the meibomian gland length. The average tortuosity, length and width of the central 8 meibomian glands were analyzed.

The CFS was performed after the instillation of fluorescein dye with impregnated narrow strips and was graded from 0 to 4 by the Baylor grading scheme [14]. Lid margin abnormalities were scored for the presence or absence of the following 4 parameters: vascular engorgement, anterior or posterior displacement of the mucocutaneous junction, irregularity of the lid margin and plugged meibomian gland orifices. The meibum expressibility score was assessed for the 15 glands on each lower eyelid and ranged from 0 to 45 [15, 16].

### Statistical analysis

Statistical analyses were performed using SPSS 22.0 (IBM, Armonk, NY). Normality distributions were tested using the Kolmogorov–Smirnov test. One-way repeated-measures analysis of variance (ANOVA) tests were used to test overall differences between visits in each group. The Bonferroni method was used in the post hoc test to evaluate the differences between the 2 visits. Two-way repeated-measures analysis of variance (ANOVA) tests were used for comparisons of overall differences between congenital/developmental cataract group and control group.

The independent-samples t-test or the Mann–Whitney U test was performed to analyze differences between congenital/developmental cataract patients and normal controls. A *P*-value less than 0.05 was considered statistically significant.

## Results

Age and sex did not differ significantly between congenital/developmental cataract patients and normal controls (*P* = 0.844 and *P* = 0.571, respectively). Data for the clinical parameters of the congenital/developmental cataract patients and the *P* values are presented in Table 1.

**Table 1:** Clinical parameters of the congenital/developmental cataract patients and normal controls

Parameters		Baseline	1 w	1 m	3 m	6 m	*P* values Overall	*P1*	*P2*	*P3*	*P4*
Ocular symptom score (0–14)	Group 1	1.70 ± 0.27	1.95 ± 0.30	1.78 ± 0.32	1.53 ± 0.31	1.00 ± 0.38	< 0.001*	0.500	0.330	0.614	0.009*
	Group 2	0.93 ± 0.25	1.50 ± 0.31	1.00 ± 0.26	0.69 ± 0.26	0.70 ± 0.30	0.009*^§^				
NIBUT-first (sec)	Group 1	13.41 ± 1.77	9.25 ± 1.27	7.28 ± 1.04	9.33 ± 1.65	11.03 ± 2.11	0.218	0.136	0.008*	0.110	0.953
	Group 2	11.47 ± 1.96	12.72 ± 1.79	11.48 ± 2.16	13.56 ± 1.96	13.68 ± 2.04	0.259^§^				
NIBUT-average (sec)	Group 1	18.18 ± 1.25	15.54 ± 1.24	12.49 ± 1.44	14.58 ± 1.62	15.80 ± 1.86	0.061	0.102	0.012*	0.050	0.953
	Group 2	13.46 ± 1.97	15.87 ± 1.78	16.16 ± 1.81	16.24 ± 1.71	16.92 ± 1.71	0.017*^§^				
CFS (0–20)	Group 1	1.61 ± 0.44	3.71 ± 0.86	3.72 ± 0.80	2.40 ± 0.79	1.92 ± 0.67	0.232	0.039*	0.040*	0.291	0.865
	Group 2	0.75 ± 0.39	1.57 ± 0.55	1.88 ± 0.47	1.77 ± 0.46	1.80 ± 0.87	0.241^§^				
TMH (mm)	Group 1	0.18 ± 0.01	0.16 ± 0.01	0.17 ± 0.02	0.18 ± 0.02	0.17 ± 0.02	0.299	0.299	0.777	0.191	0.310
	Group 2	0.16 ± 0.01	0.16 ± 0.01	0.17 ± 0.01	0.17 ± 0.01	0.18 ± 0.02	0.808^§^				
Lid margin score (0–4)	Group 1	0.95 ± 0.20	1.19 ± 0.19	1.00 ± 0.20	1.14 ± 0.77	1.23 ± 0.17	0.193	0.358	0.356	0.163	0.705
	Group 2	0.93 ± 0.34	0.85 ± 0.30	1.00 ± 0.27	1.00 ± 0.20	1.20 ± 0.20	0.449^§^				
Meiboscore (0–6)	Group 1	1.12 ± 0.31	-	1.13 ± 0.27	1.14 ± 0.22	0.85 ± 0.30	0.123	-	1.000	0.366	0.102
	Group 2	0.91 ± 0.31	-	1.08 ± 0.26	0.85 ± 0.30	0.80 ± 0.25	0.215^§^				
Meibum expressibility score (0–45)	Group 1	42.65 ± 0.70	41.88 ± 0.64	41.94 ± 0.58	41.33 ± 1.11	41.46 ± 0.75	0.024*	0.217	0.108	0.476	0.105
	Group 2	41.86 ± 1.35	42.69 ± 0.87	42.53 ± 0.72	43.00 ± 0.83	42.30 ± 1.03	0.352^§^				

*Group 1:* congenital/developmental cataract group, *Group 2:* normal control group, *NIBUT-average:* average non-invasive tear film break-up time, *NIBUT-first:* first non-invasive tear film break-up time, *CFS:* corneal fluorescein staining, *TMH:* Tear meniscus height

Overall *P* values: overall differences between baseline and visits in each group using one-way repeated-measures analysis of variance (ANOVA) tests; *P1*: 1w vs baseline; *P2*: 1 m vs baseline; *P3*: 3 m vs baseline; *P4*: 6 m vs baseline

^§^ *P* values compared between congenital/developmental cataract group and normal control group using two-way repeated-measures analysis of variance (ANOVA) tests

^*^ *P* values < 0.05

### Ocular symptoms

The ocular symptom score was significantly higher in congenital/developmental cataract patients compared to normal controls during the 5 visits (*P* = 0.009). Photophobia and vision fluctuation were the most common symptoms before surgery. Of the patients, 50% (8/16) complained of photophobia and 37.5% (6/16) complained of vision fluctuation. Meanwhile, 25% of patients (4/16) complained of ocular fatigue, which was the most common symptom at 6 months postoperatively.

### Tear film function

**Fig. 1:**
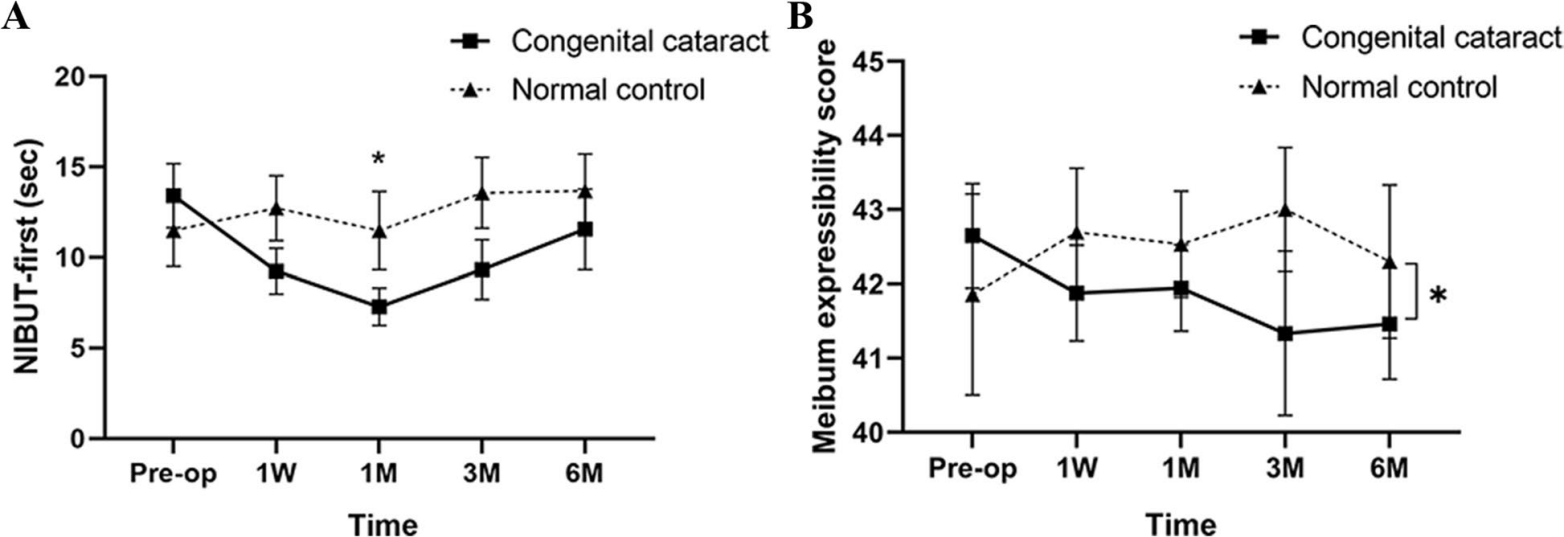
Changes in first non-invasive tear film break-up time (**A**) and meibomian gland expressibility (**B**) after congenital/developmental cataract surgery. The asterisk represents the *P* value less than 0.05

The average NIBUT was significantly lower in congenital/developmental cataract patients compared to normal controls (*P* = 0.017). Compared to baseline levels, the first NIBUT and average NIBUT decreased significantly at 1 month postoperatively (*P* = 0.008 and *P* = 0.012, respectively, Fig. 1A) and increased to baseline levels at 3 months postoperatively (*P* > 0.05). The CFS was significantly increased at 1 week and 1 month postoperatively compared to baseline levels (*P* = 0.039 and *P* = 0.040, respectively) and recovered at 3 months postoperatively. TMH did not change significantly after congenital/developmental cataract surgery compared to baseline levels of enrolled eyes (*P* > 0.05). There was also no significant difference between congenital/developmental cataract patients and normal controls for TMH and CFS (*P* > 0.05).

### Meibomian gland function

**Fig. 2:**
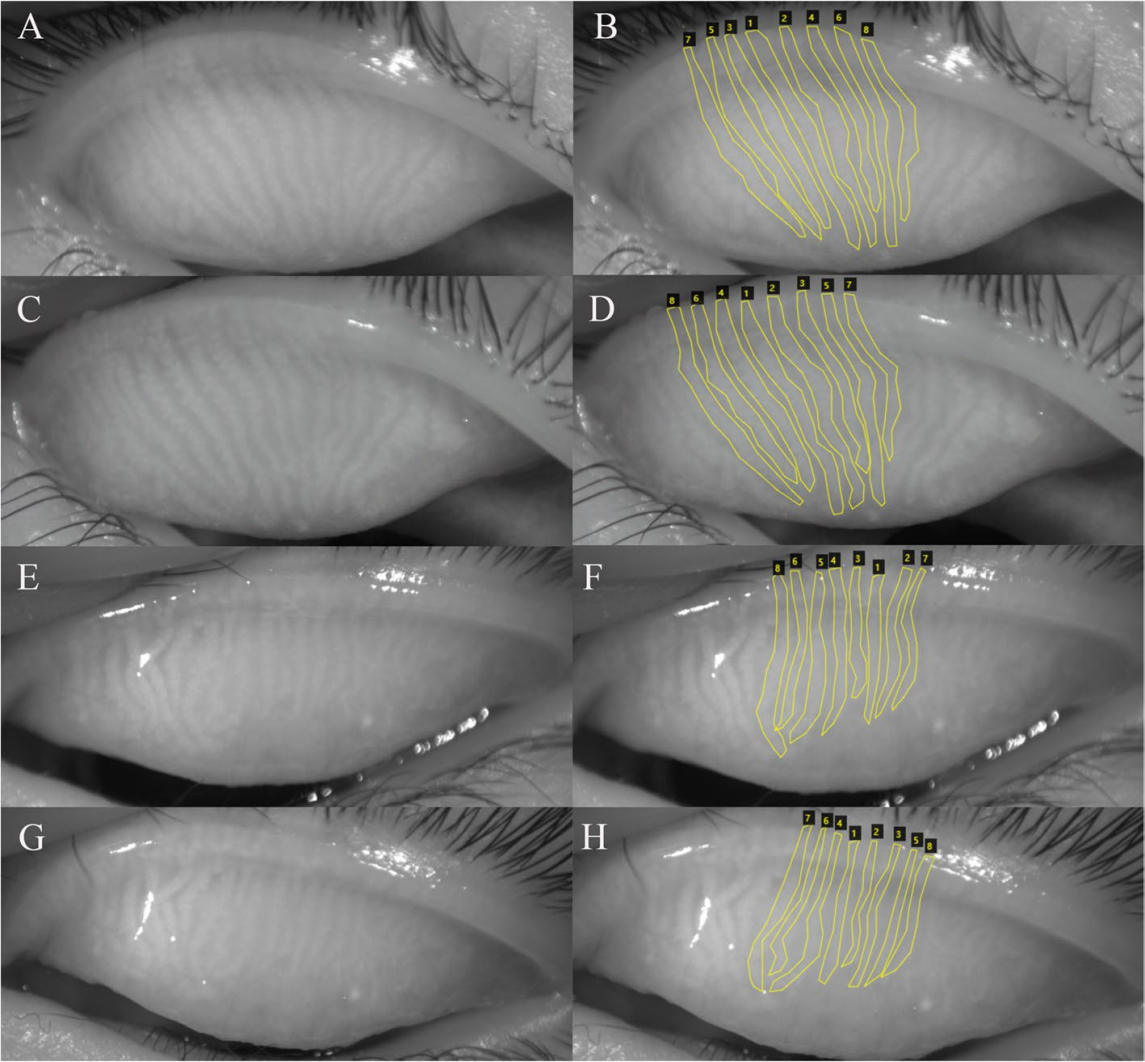
The meibomian gland images of two congenital/developmental cataract patients preoperatively and at 6 months postoperatively. **A** An original meibomian gland image of the right eye of a 12-year-old male patient preoperatively. **C** A meibomian gland image of the right eye of the 12-year-old male patient at 6 months postoperatively. **E** A meibomian gland image of the left eye of a 9-year-old male patient preoperatively. **G** A meibomian gland image of the left eye of the 9-year-old male patient at 6 months postoperatively. **B**, **D**, **F**, **H** using the “polygon region shape” function of the VIA software to line out the boundaries of the central 8 meibomian glands one by one. The average meibomian gland tortuosity of the upper eyelid for the 12-year-old male patient was 0.097 preoperatively and 0.102 at 6 months postoperatively. And it was 0.059 preoperatively and 0.069 at 6 months postoperatively

The meibum expressibility score decreased significantly during the 5 visits (*P* = 0.024, Fig. 1B), while no significant change was observed in meibomian gland tortuosity, meibomian gland width, meibomian gland area and meibomian gland length at 6 months postoperatively compared to baseline (*P* > 0.05, Fig. 2). Meiboscore and lid margin score also did not change significantly postoperatively (*P* > 0.05).

The lid margin score of the upper eyelid was significantly higher in congenital/developmental cataract patients compared to normal controls at 1 week postoperatively (*P* = 0.027). No significant difference was observed in meibomian gland tortuosity, meibomian gland width, meibomian gland area and meibomian gland length between the congenital/developmental group and normal controls preoperatively and at 6 months postoperatively (*P* > 0.05, Table 2). The meibum expressibility score, meiboscore and lid margin score also did not differ significantly between the two groups (*P* > 0.05).

**Table 2:** Meibomian gland morphological parameters of the two groups of the study population

	Congenital/developmental cataract patients	Normal controls	*P*
Meibomian gland tortuosity			
Baseline	0.23 ± 0.06	0.23 ± 0.04	0.984
6 months postoperatively	0.23 ± 0.05	0.21 ± 0.08	0.957
Meibomian gland length			
Baseline	476.08 ± 55.09	458.96 ± 33.31	0.424
6 months postoperatively	458.50 ± 64.83	429.18 ± 96.84	0.418
Meibomian gland width			
Baseline	49.24 ± 8.47	50.63 ± 3.27	0.632
6 months postoperatively	49.01 ± 6.36	48.82 ± 8.97	0.956
Meibomian gland area			
Baseline	10,686.83 ± 2484.74	10,583.24 ± 1649.94	0.917
6 months postoperatively	10,170.55 ± 1787.11	10,100.00 ± 2465.99	0.939

## Discussion

We observed changes in tear film function, meibomian gland function and meibomian gland morphology after congenital/developmental cataract surgery. Our data suggested that cataract surgery in children may influence tear film function and meibomian gland function transitorily without causing morphological changes in the meibomian glands.

Dry eye is one of the most frequent complications following adult cataract surgery. It causes ocular discomfort, fluctuating vision, and influences vision-related quality of life postoperatively owing to tear film dysfunction and MGD [17, 18]. Whether cataract surgery will influence tear film function or meibomian gland function in children is still unknown.

This study found that lid margin score increased significantly at 1 week postoperatively and began to recover at 1 month postoperatively. Vascular engorgement and anterior or posterior displacement of the mucocutaneous junction were the most common lid margin abnormalities in this study. More than 50% of eyes in this study showed vascular engorgement and anterior or posterior displacement of the mucocutaneous junction at 1 week postoperatively. Previous studies have suggested that vascular engorgement may accompany the narrowing of the meibomian gland orifices, which is a feature of early MGD [19]. Lid margin changes are also signs of inflammation, which is one of the most important mechanisms of dry eye and MGD. It is widely acknowledged that cataract surgery can induce inflammatory responses with the arrival of neutrophils and macrophages and the production of inflammatory mediators [20, 21]. The expression of the cyclooxygenase 2 (COX-2) enzyme, which catalyzes a key step in the synthesis of prostaglandin, increased at 24 h and 4 days postoperatively and reached a peak at 5 days postoperatively in lens epithelial cells [20]. Upregulated inflammatory responses may increase lid margin scores and ocular symptoms postoperatively.

This study also found that the tear film stability parameters, including the first NIBUT and average NIBUT, decreased significantly at 1 month postoperatively and recovered to baseline levels at 3 months postoperatively. And CFS increased significantly at 1 week and 1 month postoperatively and also recovered at 3 months postoperatively. It was widely agreed that an unstable tear film has a pivotal role in the mechanism of dry eye, leading to symptoms and visual impairments [18]. Of the participants, 40% (6/15) had their first NIBUT lower than 5 s at 1 month postoperatively. In adults, TBUT decreased significantly at 1 and 3 months after cataract surgery, which was in accordance with this study [17]. Inflammation and topical medications may contribute to the unstable tear film and CFS postoperatively. Topical medications were used for all patients until 1 month postoperatively, which may have further affected tear film stability during the early postoperative period. The benzalkonium chloride in the eye drops may cause damage to conjunctival and corneal epithelial cells and influence the tear film [22]. On the other hand, topical antibiotics and steroid use may also help to treat the inflammation and improve clinical parameters.

In our study, detailed changes in the meibomian glands were assessed, including the tortuosity, width, area and length. Of the participants, 47.06% had meibomian gland atrophy, and 21.43% had meibomian gland tortuosity. Previous studies have observed that meibomian gland morphology changes, including atrophy, start early in life [23]. Moreover, meibomian gland morphologic changes can be an early and sensitive indicator of MGD [24, 25]. A study by Shirakawa R et al. found that no significant meibomian gland shortening or dropout was observed in children under 3 years of age, [26] while in children aged 4 to 17 years, 42% had some evidence of meibomian gland atrophy [23]. Another study found that 45.5% of asymptomatic children between 7 and 14 years old had an MG deficiency between 20 and 30% [8]. However, our study did not find significant meibomian gland morphological changes within 6 months after cataract surgery compared to baseline levels and normal controls. This result was in accordance with the study in adults after cataract surgery [17].

The TMH did not change significantly after cataract, which indicated a minor change in tear volume. A previous study also found that lower tear meniscus height, depth and area did not change significantly within 3 months after cataract surgery in adults [17].

The first limitation of this study was that the sample size was not large enough for further analysis. Second, a longer follow-up may be needed to observe long-term changes in the meibomian glands and tear films. Moreover, placebo eye drops with the same benzalkonium chloride should be applied for normal controls. And the correlation between ocular surface and duration of medications should also be investigated.

## Conclusion

Tear film stability and meibomian gland function change transiently after congenital/developmental cataract surgery without meibomian gland morphological changes.

## Data Availability

The data that support the findings of this study are available from the corresponding author, Y.L., upon reasonable request.

## Abbreviations

MGD: Meibomian gland dysfunction
TMH: Tear meniscus height
NIBUT: Noninvasive breakup time
CFS: Corneal fluorescein staining
BMI: Body mass index
ANOVA: Analysis of variance
COX-2: Cyclooxygenase 2

## References

[1] Self JE, Taylor R, Solebo AL, Biswas S, Parulekar M, Dev Borman A (2020). Cataract management in children: a review of the literature and current practice across five large UK centres. Eye.

[2] Mohammadpour M, Shaabani A, Sahraian A, Momenaei B, Tayebi F, Bayat R (2019). Updates on managements of pediatric cataract. Journal of Current Ophthalmology.

[3] Sitompul R, Sancoyo GS, Hutauruk JA, Gondhowiardjo TD (2008). Sensitivity change in cornea and tear layer due to incision difference on cataract surgery with either manual small-incision cataract surgery or phacoemulsification. Cornea.

[4] Kohlhaas M (1998). Corneal sensation after cataract and refractive surgery. J Cataract Refract Surg.

[5] Shoss BL, Tsai LM (2013). Postoperative care in cataract surgery. Curr Opin Ophthalmol.

[6] Stapleton F, Alves M, Bunya VY, Jalbert I, Lekhanont K, Malet F (2017). TFOS DEWS II epidemiology report. Ocul Surf.

[7] Gupta PK, Venkateswaran N, Heinke J, Stinnett SS (2020). Association of meibomian gland architecture and body mass index in a pediatric population. Ocul Surf.

[8] Zhao Y, Chen S, Wang S, Chen Y, Li J, Fu Y (2018). The significance of meibomian gland changes in asymptomatic children. Ocul Surf.

[9] Al Hayouti H, Daniel M, Hingorani M, Calder V, Dahlmann Noor A (2019). Meibography and corneal volume optical coherence tomography to quantify damage to ocular structures in children with blepharokeratoconjunctivitis. Acta Ophthalmol.

[10] Tichenor AA, Ziemanski JF, Ngo W, Nichols JJ, Nichols KK (2019). Tear film and meibomian gland characteristics in adolescents. Cornea.

[11] Arita R, Itoh K, Inoue K, Amano S (2008). Noncontact infrared meibography to document age-related changes of the meibomian glands in a normal population. Ophthalmology.

[12] Dutta A, Zisserman A. The VIA annotation software for images, audio and video. In: Proceedings of the 27th ACM International Conference on Multimedia (MM ’19). New York: Association for Computing Machinery; 2019. p. 2276–79.

[13] Lin X, Fu Y, Li L, Chen C, Chen X, Mao Y (2020). A novel quantitative index of meibomian gland dysfunction, the meibomian gland tortuosity. Transl Vis Sci Technol.

[14] De Paiva CS, Pflugfelder SC (2004). Corneal epitheliopathy of dry eye induces hyperesthesia to mechanical air jet stimulation. Am J Ophthalmol.

[15] Cochener B, Cassan A, Omiel L (2018). Prevalence of meibomian gland dysfunction at the time of cataract surgery. J Cataract Refract Surg.

[16] Lane SS, DuBiner HB, Epstein RJ, Ernest PH, Greiner JV, Hardten DR (2012). A new system, the LipiFlow, for the treatment of meibomian gland dysfunction. Cornea.

[17] Han KE, Yoon SC, Ahn JM, Nam SM, Stulting RD, Kim EK (2014). Evaluation of dry eye and meibomian gland dysfunction after cataract surgery. Am J Ophthalmol.

[18] Tsubota K, Yokoi N, Shimazaki J, Watanabe H, Dogru M, Yamada M (2017). New perspectives on dry eye definition and diagnosis: a consensus report by the Asia dry eye society. Ocul Surf.

[19] Tomlinson A, Bron AJ, Korb DR, Amano S, Paugh JR, Pearce EI (2011). The international workshop on meibomian gland dysfunction: report of the diagnosis subcommittee. Invest Ophthalmol Vis Sci.

[20] Jiang J, Shihan MH, Wang Y, Duncan MK (2018). Lens epithelial cells initiate an inflammatory response following cataract surgery. Invest Ophthalmol Vis Sci.

[21] Sutu C, Fukuoka H, Afshari NA (2016). Mechanisms and management of dry eye in cataract surgery patients. Curr Opin Ophthalmol.

[22] Goldstein MH, Silva FQ, Blender N, Tran T, Vantipalli S (2022). Ocular benzalkonium chloride exposure: problems and solutions. Eye (Lond).

[23] Gupta PK, Stevens MN, Kashyap N, Priestley Y (2018). Prevalence of meibomian gland atrophy in a pediatric population. Cornea.

[24] Adil MY, Xiao J, Olafsson J, Chen X, Lagali NS, Ræder S (2019). Meibomian gland morphology is a sensitive early indicator of meibomian gland dysfunction. Am J Ophthalmol.

[25] Wu Y, Li H, Tang Y, Yan X. Morphological evaluation of meibomian glands in children and adolescents using noncontact infrared meibography. J Pediatr Ophthalmol Strabismus. 2017;54(2):78–83.

[26] Shirakawa R, Arita R, Amano S (2013). Meibomian gland morphology in Japanese infants, children, and adults observed using a mobile pen-shaped infrared meibography device. Am J Ophthalmol.

